# The systematics of *Echinorhynchus* Zoega in Müller, 1776 (Acanthocephala, Echinorhynchidae) elucidated by nuclear and mitochondrial sequence data from eight European taxa

**DOI:** 10.3897/zookeys.484.9132

**Published:** 2015-02-26

**Authors:** Matthew T. Wayland, Jouni K. Vainio, David I. Gibson, Elisabeth A. Herniou, D. Timothy J. Littlewood, Risto Väinölä

**Affiliations:** 1 Department of Zoology, University of Cambridge, Downing Street, Cambridge, CB2 3EJ, United Kingdom; 2 Department of Biosciences, P.O. Box 65 (Viikinkaari 1), FIN-00014, University of Helsinki, Helsinki, Finland; 3 Department of Life Sciences, Natural History Museum, London, SW7 5BD, United Kingdom; 4 Institut de Recherche sur la Biologie de l’Insecte, UMR CNRS 7261, Université François Rabelais de Tours, Faculté des Sciences et Techniques, Avenue Monge, Parc Grandmont, 372000, Tours, France; 5 Finnish Museum of Natural History, POB 17, FIN-00014, University of Helsinki, Helsinki, Finland

**Keywords:** Acanthocephala, *Echinorhynchus bothniensis*, *Echinorhynchus brayi*, *Echinorhynchus cinctulus*, *Echinorhynchus gadi*, *Echinorhynchus salmonis*, *Echinorhynchus truttae*, *Acanthocephalus lucii*, phylogeny, molecular phylogeny, taxonomy, parasite, systematics, zoogeography

## Abstract

The acanthocephalan genus *Echinorhynchus* Zoega in Müller, 1776 (*sensu* Yamaguti 1963) is a large and widespread group of parasites of teleost fish and malacostracan crustaceans, distributed from the Arctic to the Antarctic in habitats ranging from freshwaters to the deep-sea. A total of 52 species are currently recognised based on the conventional morphological species concept; however, the true diversity in the genus is masked by cryptic speciation. The considerable diversity within *Echinorhynchus* is an argument for subdividing the genus if monophyletic groups with supporting morphological characters can be identified. With this objective in mind, partial sequences of two genes with different rates of evolution and patterns of inheritance (nuclear 28S rRNA and mitochondrial cytochrome c oxidase subunit I) were used to infer the phylogenetic relationships among eight taxa of *Echinorhynchus*. These included representatives of each of three genus group taxa proposed in a controversial revision of the genus based on cement gland pattern, namely *Echinorhynchus* (*sensu stricto*), *Metechinorhynchus* Petrochenko, 1956 and *Pseudoechinorhynchus* Petrochenko, 1956. These groupings have previously been rejected by some authorities, because the diagnostic character is poorly defined; this study shows that *Echinorhynchus* (*sensu stricto*) and *Metechinorhynchus* are not natural, monophyletic groups. A revision of *Echinorhynchus* will require tandem molecular phylogenetic and morphological analyses of a larger sample of taxa, but this study has identified two morhological characters that might potentially be used to define new genera. The estimated phylogeny also provides insight into the zoogeographical history of *Echinorhynchus* spp. We postulate that the ancestral *Echinorhynchus* had a freshwater origin and the genus subsequently invaded the sea, probably several times. The freshwater taxa of the *Echinorhynchus bothniensis* Zdzitowiecki & Valtonen, 1987 clade may represent a reinvasion of freshwater by one or more ancestral marine species.

## Introduction

**Figure 1.**
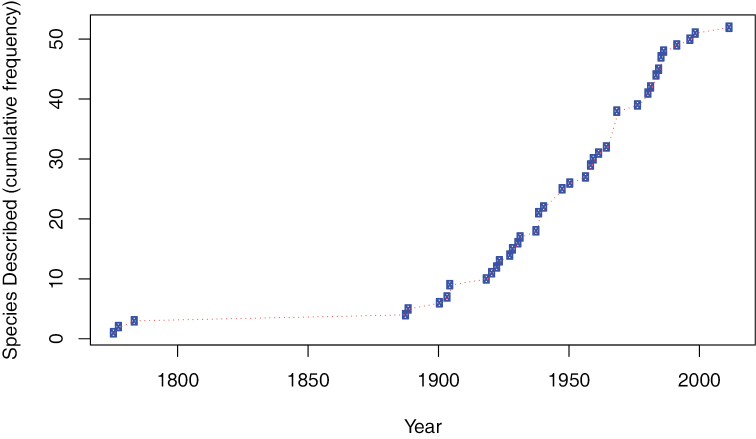
Historical record of species discovery in *Echinorhynchus*. Recognised diversity, as measured by the cumulative number of described taxa, plotted against time. Only species recognised by Amin (2013) are included.

The acanthocephalan genus *Echinorhynchus* Zoega in Müller, 1776 (*sensu* Yamaguti 1963) is a large and widespread group of parasites of teleost fish and malacostracan crustaceans, distributed from the Arctic to the Antarctic in diverse aquatic environments, including mountain streams, rivers, lakes, estuaries, coastal marine waters and the deep-sea. Over the last 125 years the number of described taxa has steadily increased (Fig. 1), a trend which may well continue, since many, if not most potential hosts (particularly from the deep-sea) have yet to be surveyed for parasites. A total of 52 species of *Echinorhynchus* were recognised in the most recent classification of the Acanthocephala (Amin 2013); however, the morphological species concept used to define these taxa masks the true diversity in the genus. Allozyme electrophoresis has revealed cryptic speciation within the marine *Echinorhynchus gadi* Zoega in Müller, 1776 and the freshwater *Echinorhynchus bothniensis* Zdzitowiecki & Valtonen, 1987 (see Väinölä et al. 1994). It is reasonable to assume that other taxa may also comprise sibling species. In addition to demonstrating previously unrecognised diversity in *Echinorhynchus*, allozyme electrophoresis also showed marked genetic divergence between the species of the *Echinorhynchus gadi* complex and *Echinorhynchus salmonis* Müller, 1784 (genetic identity ≈ 0), suggesting that the genus represents “an evolutionary unit deeper and wider than genera in most other animal groups” (Väinölä et al. 1994).

**Figure 2.**
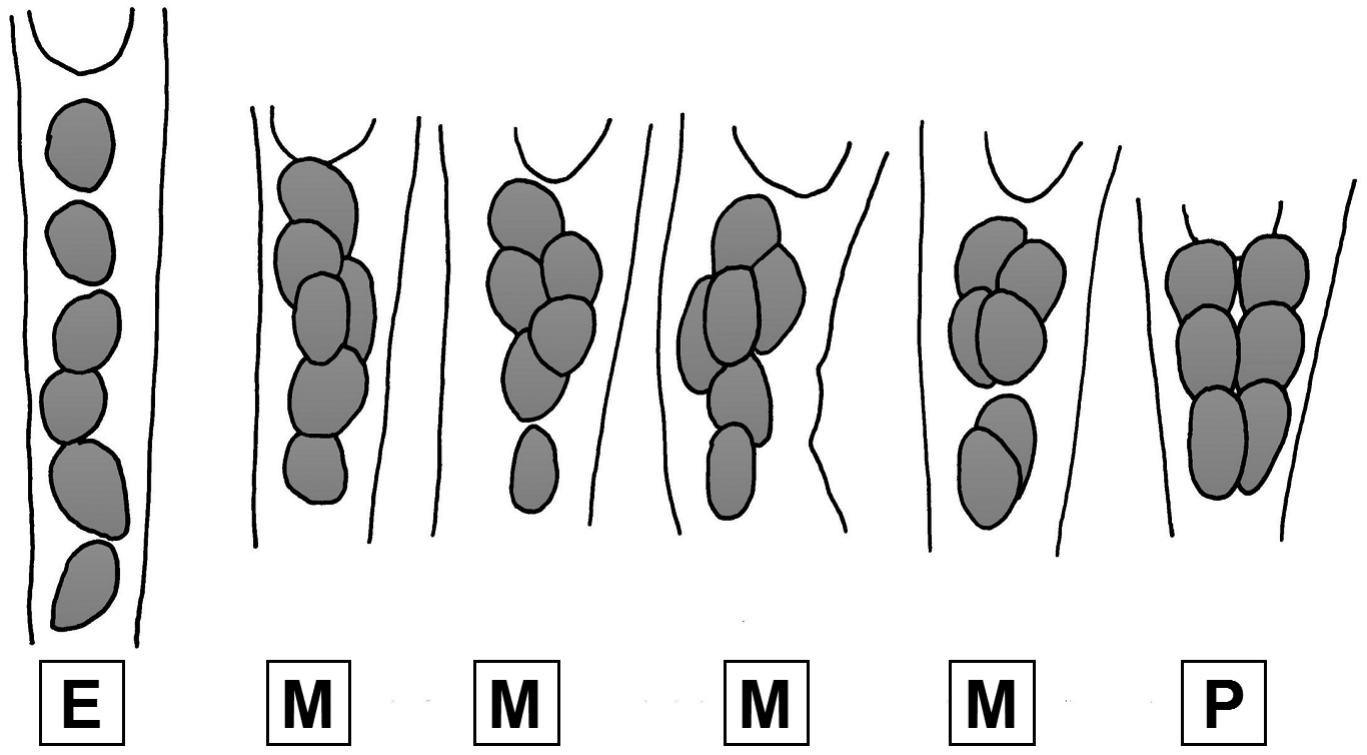
Cement gland arrangements of the genera recognised by Petrochenko (1956). E. *Echinorhynchus*. M. *Metechinorhynchus*. P. *Pseudoechinorhynchus*.

Given the species diversity and genetic divergence within *Echinorhynchus*, it would be useful to split the genus if monophyletic groups with supporting morphological characters can be identified. Petrochenko (1956) attempted to revise this genus on the basis of cement gland pattern, which he considered to be a “fairly constant” taxonomic character. He amended *Echinorhynchus* (type-species: *Echinorhynchus gadi*) to include only those worms which have their cement glands situated along the mid-line like a “string of beads”. At the same time, he erected two new genera, *Pseudoechinorhynchus* Petrochenko, 1956 (type-species: *Pseudoechinorhynchus clavula* (Dujardin, 1845)) for acanthocephalans displaying three regular pairs of cement glands and *Metechinorhynchus* Petrochenko, 1956 (type-species: *Metechinorhynchus salmonis*) for worms having cement glands arranged in no definite pattern (Fig. 2). Petrochenko’s three genera appeared to have the attractive property of being associated with the habitat of the acanthocephalan’s hosts: species of *Echinorhynchus* are parasites of marine fish, whereas species of *Metechinorhynchus* and *Pseudoechinorhynchus* were thought to be typically parasites of freshwater fish.

Golvan (1969) initially accepted Petrochenko’s classification with only minor amendments. However, he later relegated *Pseudoechinorhynchus* and *Metechinorhynchus* to the status of subgenera of *Echinorhynchus* (*sensu lato*) (see Golvan 1994). Huffman and Kliever (1977) felt unable to place a new species of *Echinorhynchus* (*sensu lato*) in Petrochenko’s system. Most specimens of *Echinorhynchus canyonensis* Huffman & Kliever, 1977 conformed to the diagnosis of *Metechinorhynchus*, but some displayed the moniliform cement gland pattern of *Echinorhynchus* (*sensu stricto*). Huffman and Kliever considered Petrochenko’s genera ill-defined and *Metechinorhynchus* to be particularly ambiguous, a view shared by Amin and Redlin (1980), who found that male *Echinorhynchus salmonis* (type-species of *Metechinorhynchus*) frequently exhibited the evenly paired cement glands characteristic of *Pseudoechinorhynchus*. Both pairs of authors concurred with Yamaguti (1963) in regarding *Pseudoechinorhynchus* and *Metechinorhynchus* to be junior synonyms of *Echinorhynchus*. In this paper, *Echinorhynchus* will be used to refer to the broad concept of the genus *sensu* Yamaguti (1963), unless otherwise stated.

Although molecular systematics have revealed that species of *Echinorhynchus* show a degree of genetic divergence that would indicate a generic division, such a division would not produce taxa concordant with Petrochenko’s system (Väinölä et al. 1994). If *Echinorhynchus bothniensis* was to be classified under Petrochenko’s scheme, it would be placed in *Metechinorhynchus*, since males exhibit no definite cement gland pattern (Zdzitowiecki and Valtonen 1987). However, phylogenetic analysis of allozyme data indicated that *Echinorhynchus bothniensis* has a much closer affinity to the *Echinorhynchus gadi* (type-species of *Echinorhynchus* (*sensu stricto*)) complex than to *Echinorhynchus salmonis* (type-species of *Metechinorhynchus*), indicating that *Metechinorhynchus* would be paraphyletic.

A further problem for Petrochenko’s classification is the taxonomic status of *Pseudoechinorhynchus clavula*, his type-species for *Pseudoechinorhynchus*. When Petrochenko published his classification, two morphologically distinct species were conflated under the specific binomen *Echinorhynchus clavula* Dujardin, 1845. Dujardin’s original description did not include drawings and lacked sufficient detail for the taxon to be reliably identified by other workers. Subsequently, Lühe (1911) made a redescription of the species with figures, based on a collection of acanthocephalans which conformed to Dujardin’s incomplete description, but were not in fact conspecific. Lühe’s more detailed description became the reference for determining this taxon.

The incompatibility between *Echinorhynchus clavula* Dujardin and *Echinorhynchus clavula* Dujardin *sensu* Lühe (1911) became apparent when Grabda-Kazubska and Chubb (1968) compared acanthocephalans determined as *Echinorhynchus clavula* from the British Isles with those from Poland which fitted the description given by Lühe (1911). Both groups conformed to the diagnosis of the subfamily Echinorhynchinae Cobbold, 1879, but they differed from each other in a key generic character, the position of the nerve ganglion in the proboscis receptacle. In the acanthocephalans from the British Isles, the nerve ganglion was situated at the base of the proboscis receptacle, placing this group in the genus *Acanthocephalus* Koelreuther, 1771. However, in the Polish sample, the nerve ganglion was situated mid-way along the proboscis receptacle, as is characteristic of species of *Echinorhynchus*. Through reference to Dujardin’s unpublished drawings of *Echinorhynchus clavula*, which indicated a basal position for the nerve ganglion in the proboscis receptacle, Grabda-Kazubska and Chubb (1968) were able to conclude that the material from the British Isles conformed to the original concept of *Echinorhynchus clavula* and that the correct name of this taxon was *Acanthocephalus clavula* (Dujardin, 1845). These authors asserted that *Echinorhynchus clavula* Dujardin *sensu* Lühe should remain in the genus *Echinorhynchus* under the name of *Echinorhynchus borealis* von Linstow, 1901. However, since this latter name is pre-occupied, *Echinorhynchus borealis* von Linstow, 1901 is now considered a synonym of *Echinorhynchus cinctulus* Porta, 1905 (see Golvan 1994, Amin 2013). Petrochenko (1956) used Lühe’s description of *Echinorhynchus clavula* in his classification and therefore *Echinorhynchus cinctulus* would be the type-species of *Pseudoechinorhynchus*, if this genus was to be recognised as a valid taxon.

Further attempts at revising *Echinorhynchus* should be underpinned by evidence of the phylogenetic relationships of its constituent taxa. To this end we have used sequences from two genes with different patterns of inheritance and different rates of evolutionary change (28S rRNA and cytochrome c oxidase subunit I) to reconstruct a phylogeny for nine populations of *Echinorhynchus*, representing eight distinct biological taxa (Table 1). In addition to resolving taxonomic problems, phylogenetic analyses of the relationships of *Echinorhynchus* species present the best means of understanding the zoogeography of the group.

**Table 1.** Sample information.

Species	Host	Locality	Date collected	Genus *sensu* Petrochenko (1956)	Environment	GenBank # (28S rDNA / COI)	Voucher specimens
*Acanthocephalus lucii* (outgroup)	*Perca fluviatilis* (L.) (Percidae)	Lake, Bleasby, Nottinghamshire, UK	4/06/1997	*Acanthocephalus*	Freshwater	KM656148 / KP261016	BM(NH) 2002.2.4.284–292
*Echinorhynchus bothniensis*	*Osmerus eperlanus* (L.) (Osmeridae)	Lake Keitele, central Finland	10/10/1996	*Metechinorhynchus*	Freshwater	KM656146 / KP261018	BM(NH) 2002.2.4.102–122
*Echinorhynchus 'bothniensis'*	*Platichthys flesus* (L.) (Pleuronectidae) *Mysis segerstralei* Audzijonyte & Väinölä (Mysidae)*	Lake Pulmankijärvi, northern Finland	11/06/1990	*Echinorhynchus*	Freshwater	KM656143 / KP261019	NA
*Echinorhynchus brayi*	*Pachycara crassiceps* (Roule) (Zoarcidae)	Porcupine Seabight, 49°49.9'N, 13°08.2'W, depth 2,444 m	13/08/1997	*Metechinorhynchus*	Marine, deep-sea	KM656151 / KP261015	BM(NH) 1997.12.8.3 (holotype); BM(NH) 1997.12.8.4–28
*Echinorhynchus cinctulus* (= *Echinorhynchus borealis*)	*Lota lota* (L.) (Lotidae)	Kuopio, Finland	15/10/1996	*Pseudoechinorhynchus*	Freshwater	KM656142 / KP261014	BM(NH) 2002.2.4.123–131
*Echinorhynchus gadi* sp. I	*Gadus morhua* L. (Gadidae)	Baltic Sea, off Tvärminne, Hanko	21/10/1992	*Echinorhynchus*	Marine	KM656144 / KP261022	BM(NH) 2002.2.4.90–101
*Echinorhynchus gadi* sp. I	*Gadus morhua*	Mys Kartesh, Gulf of Kandalaksha, White Sea	31/08/1994–2/09/1994	*Echinorhynchus*	Marine	KM656150 / KP261021	NA
*Echinorhynchus gadi* sp. III	*Gadus morhua*	Mys Kartesh, Gulf of Kandalaksha, White Sea	31/08/1994–2/09/1994	*Echinorhynchus*	Marine	KM656149 / KP261020	NA
*Echinorhynchus salmonis*	*Coregonus lavaretus* (L.) (Salmonidae)	Bothnian Bay, Baltic Sea	27/08/1996	*Metechinorhynchus*	Freshwater	KM656145 / KP261017	BM(NH) 2002.2.4.132–226
*Echinorhynchus truttae*	*Salmo trutta* L. (Salmonidae)	Loch Walton Burn, River Carron catchment, central Scotland (National Grid Reference NS 668 865)	24/06/1996	*Metechinorhynchus*	Freshwater	KM656147 / KP261013	BM(NH) 2002.2.4.264–275

* Acanthocephalans from *Platichthys flesus* and *Mysis segerstralei* were the source of the 28S rDNA and COI sequences, respectively.

## Material and methods

### Taxa sampled

Collection data for the samples are provided in Table 1. This section provides a description of the samples analyzed, summarized by nominal taxon. In order to gain insight into the zoogeography of *Echinorhynchus*, samples were selected to include taxa from a range of aquatic environments, including: both lotic and lentic freshwaters, coastal marine waters and the deep-sea. All three of Petrochenko’s genera are represented in the material, including the type-species of each. Furthermore, the samples include four taxa of *Metechinorhynchus*, so that the apparent paraphyly of this taxon (Väinölä et al. 1994) can be tested. The samples also represent a range of different levels in the systematic hierarchy from conspecific populations to taxa displaying strong genetic divergence for a congeneric comparison, according to the allozyme study of Väinölä et al. (1994). Individual molecular markers are generally suitable for phylogeny reconstruction at a particular level in the systematic hierarchy (Avise 1994). Consequently, the current study aims to provide some indication of the phylogenetic resolution provided by 28S rRNA and COI genes in terms of acanthocephalan systematics, which should inform the planning of future phylogenetic studies on this group of helminths.

*Echinorhynchus bothniensis* Zdzitowiecki & Valtonen, 1987 is known from fresh- and brackish-water environments of Northern Fennoscandia. Based on molecular differences, it may be further subdivided into two allopatric taxa (Väinölä et al. 1994). One of them occurs in the Bothnian Bay of the Baltic Sea (type-locality) and Lake Keitele, central Finland, where it uses *Osmerus eperlanus* (L.) as a definitive host and *Mysis relicta* Lovén (= *Mysis relicta* sp. I *sensu* Väinölä 1986) as an intermediate host. The second one is found in Lake Pulmankijärvi, northern Finland, and was designated *Echinorhynchus ‘bothniensis’* (Väinölä et al. 1994). The definitive hosts of *Echinorhynchus ‘bothniensis’* include *Coregonus lavaretus* (L.), *Platichthys flesus* (L.) and *Salvelinus alpinus* (L.). *Mysis segerstralei* Audzijonytė and Väinölä 2005 (= *Mysis relicta* sp. III *sensu* Väinölä 1986) is the intermediate host (Väinölä et al. 1994). Usage of a mysid intermediate host is rare in members of *Echinorhynchus*, being reported for only one other species, the Nearctic *Echinorhynchus leidyi* Van Cleave, 1924 (Prychitko and Nero 1983, Wolff 1984); all other known life-cycles of *Echinorhynchus* spp. involve amphipod intermediate hosts. *Echinorhynchus bothniensis* and *Echinorhynchus ‘bothniensis’* cannot be consistently distinguished by morphology alone (Wayland 2013), but the range of their cement gland patterns, like those of many other species in the genus, straddle the generic boundaries proposed by Petrochenko (1956). Most specimens of *Echinorhynchus bothniensis* conform to the diagnosis of *Metechinorhynchus*, whereas the majority of specimens of *Echinorhynchus ‘bothniensis’* conform to the diagnosis of *Echinorhynchus* (*sensu stricto*).

*Echinorhynchus brayi* Wayland, Sommerville & Gibson, 1999 was described from *Pachycara crassiceps* (Roule) (Zoarcidae) collected from the Porcupine Seabight at a depth of 2,444 metres (Wayland et al. 1999). The samples used in this study were collected from the same host (infrapopulation) as the type-specimens. Similarities in morphology and common usage of a deep-sea zoarcid definitive host suggest a phylogenetic affinity to the Pacific *Echinorhynchus canyonensis* Huffman & Kliever, 1977. The intermediate host of *Echinorhynchus brayi* is not known, but may well be an amphipod, given that this crustacean order is both the typical intermediate host of *Echinorhynchus* spp. and an important part of the diet of *Pachycara crassiceps*. Allozyme electrophoresis has previously shown that *Echinorhynchus brayi* is genetically divergent from the *Echinorhynchus gadi* complex, sharing not one allozyme at any of seven surveyed loci (Wayland et al. 2005). *Echinorhynchus brayi* displays the cement gland arrangement characteristic of *Metechinorhynchus* (Table 2).

**Table 2.** Cement gland arrangement in male *Echinorhynchus* spp. Notation for cement gland pattern from Shostak et al. (1986): A, clumped, three even pairs; B, clumped, three staggered pairs; C, chain-like, two pairs and two singles; D, chain-like, one pair and four singles; E, chain-like, six singles. Only specimens with six cement glands were used. Data sources: *Echinorhynchus bothniensis*, *Echinorhynchus ‘bothniensis‘* and *Echinorhynchus truttae* (Wayland 2013); *Echinorhynchus brayi*, *Echinorhynchus gadi* and *Echinorhynchus salmonis* (Wayland 2002); *Echinorhynchus cinctulus* (Grabda-Kazubska and Ejsymont 1969).

Species	A	B	C	D	E
*Echinorhynchus bothniensis*	0	1 (5.3%)	4 (21.1%)	10 (52.6%)	4 (21.1%)
*Echinorhynchus 'bothniensis'*	0	0	0	4 (44.4%)	5 (55.6%)
*Echinorhynchus brayi*	1 (8%)	7 (54%)	3 (23%)	2 (15%)	0
*Echinorhynchus cinctulus*	218 (100%)	0	0	0	0
*Echinorhynchus gadi*	0	0	0	3 (8%)	34 (92%)
*Echinorhynchus truttae*	0	1 (3%)	16 (53%)	13 (43%)	0
*Echinorhynchus salmonis*	6 (37.5%)	10 (62.5%)	0	0	0

As explained in the Introduction, *Echinorhynchus cinctulus* Porta, 1905 is the correct name for the type-species of Petrochenko’s genus *Pseudoechinorhynchus* that has commonly been referred to as *Echinorhynchus borealis* Linstow. This species is found in fresh and oligohaline waters of the Palaearctic (Grabda-Kazubska and Ejsymont 1969). The burbot *Lota lota* (L.) (Lotidae) is the usual definitive host, but it has been found in a systematically diverse range of fishes (Grabda-Kazubska and Ejsymont 1969). Intermediate hosts of *Echinorhynchus cinctulus* are the amphipods: *Gammarus pulex* L. (see Nybelin 1923), *Pallaseopsis quadrispinosa* (G.O. Sars, 1867) (see Valtonen and Crompton 1990) and *Monoporeia affinis* (Lindström, 1855) (see Bauer 1953).

*Echinorhynchus gadi* Zoega in Müller, 1776, the type-species of *Echinorhynchus*, is the most frequently reported acanthocephalan from fish of the North Atlantic and North Pacific Oceans (Gibson 2001). The definitive host spectrum is broad, and numerous amphipod crustacean species have been reported as intermediate hosts (Marcogliese 1994). Using allozyme electrophoresis Väinölä et al. (1994) demonstrated that *Echinorhynchus gadi* from gadid fish of the northeast Atlantic comprises at least three, partly sympatric, sibling species, designated species I-III. Species I was present in all regions sampled, namely the northern Baltic, North Sea and Norwegian Sea. Species II was found in the North Sea and species III in the Norwegian Sea. Subsequently, both species I and III were also identified in the Gulf of Kandalaksha, White Sea (Väinölä, unpubl.). In the present study, we analyze allozymically identified samples from the Baltic and White Sea populations of species I and the White Sea population of species III. A later allozyme study also detected two sympatric sibling species of *Echinorhynchus gadi* in gadid fish from the North Sea (termed species A and B) and further demonstrated that they could be distinguished on the basis of subtle differences in hook morphometrics (Wayland et al. 2005). Morphological similarity suggested that species A of Wayland et al. (2005) is probably conspecific with species I of Väinölä et al. (1994). A more recent study of *Echinorhynchus gadi* from Atlantic cod *Gadus morhua* L. did not find variation among eight North Atlantic and Arctic populations in the slowly evolving 18S rRNA sequence marker (Sobecka et al. 2011).

*Echinorhynchus salmonis* Müller, 1784 is the type-species of Petrochenko’s (1956) genus *Metechinorhynchus*. This is a fresh and brackish water species distributed throughout much of the Holarctic. Salmoniform fishes are the usual definitive host of this parasite, but it can develop to sexual maturity in a systematically diverse range of fish hosts (Valtonen and Crompton 1990). The amphipod intermediate hosts include species of *Gammarus* Fabricius, 1775, *Pallaseopsis* Kamaltynov & Väinölä, 2002, *Monoporeia* Bousfield, 1989 and *Diporeia* Bousfield, 1989 (e.g. Valtonen 1980, Measures and Bossé 1993). The population from which the sample used in this study was taken was characterized morphologically by Wayland et al. (2004).

*Echinorhynchus truttae* Schrank, 1788 is another common parasite of salmonid fishes in northern Europe. In the original description of *Echinorhynchus bothniensis*, Zdzitowiecki and Valtonen (1987) distinguished their new taxon from *Echinorhynchus truttae* on the basis that it had a shorter proboscis and much longer eggs. A subsequent analysis of morphological variation in these taxa demonstrated that *Echinorhynchus truttae* cannot be distinguished from *Echinorhynchus bothniensis* or *Echinorhynchus ‘bothniensis’* on the basis of proboscis length, egg length or any other conventional morphological character (Wayland 2013). However, *Echinorhynchus truttae* can be discriminated from the *Echinorhynchus bothniensis* group using multivariate analysis of hook morphometrics (Wayland 2013), as applied by the Proboscis Profiler tool (Wayland 2010). The amphipod intermediate hosts include *Gammarus fossarum* Koch, 1836 (see Van Maren 1979) and *Gammarus pulex* L. (see Lühe 1911). Petrochenko (1956) assigned *Echinorhynchus truttae* to *Metechinorhynchus*. The sample was taken from a population which has been studied morphologically (Wayland 2013).

In order to root the phylogenetic trees, sequence data were also determined from *Acanthocephalus lucii* (Müller, 1776), another member of the subfamily Echinorhynchinae. *Acanthocephalus* and *Echinorhynchus* appear to be closely related genera discriminated on the basis of only one morphological character, the position of the nerve ganglion or “brain”, which is situated at the base of the proboscis receptacle in *Acanthocephalus* but mid-way along the receptacle in *Echinorhynchus* (see Petrochenko 1956). Moreover, molecular phylogenies for the Acanthocephala demonstrate an affinity between these two genera (García-Varela and Nadler 2005, 2006). The principal definitive host of *Acanthocephalus lucii* is the perch *Perca fluviatilis* L. (see Brattey 1988) and its intermediate host is the isopod *Asellus aquaticus* L. (see Andryuk 1979, Brattey 1983). The cement glands of *Acanthocephalus lucii* are typically arranged in pairs (Petrochenko 1956).

### Sample collection and DNA extraction

All acanthocephalans were washed in saline and then fixed in 90–100% alcohol immediately after collection, or alternatively frozen in liquid nitrogen and stored at -80 °C. Single specimens of each sample were used for the sequencing of each gene, but different individuals were analyzed for the different genes (in different laboratories). The anterior ends of the worms were removed before DNA extraction to avoid contamination of the samples with any host tissue attached to the proboscis. For the 28S analysis, individual acanthocephalans were washed in TE, ground in 150 µl TE (pH 8.0), 0.5% SDS, and digested overnight with the addition of 6 µl proteinase K (10 mg ml^-1^) at 37 °C. DNA was phenol-chloroform extracted and precipitated for 15 minutes at -20 °C with 0.1 vol. sodium acetate, at pH 5.0, and 2.5 vols 100% ethanol. DNA pellets were washed in 70% ethanol, dried, resuspended in TE (pH 8.0) and stored at -20 °C. Spectrophotometry was used to estimate the concentration of nucleic acids. Alternatively, for the COI data set, the CTAB extraction protocol of Doyle and Dickson (1987) was used.

### DNA amplification and sequencing

For most taxa, a c.1,600 base-pair segment of the 28S rRNA gene spanning variable regions D1 to D6 was amplified using the primers LSU5 (5´-TAGGTCGACCCGCTGAAYTTAAGCA-3) and LSUD6-3 (5´-GGAACCCTTCTCCACTTCAGTC-3´) (Littlewood et al. 2000). For sequencing, these two amplification primers along with three internal primers were used (ECD2: 5´-CCTTGGTCCGTGTTTCAAGACGGG-3´, 900F: 5´-CCGTCTTGAAACACGGACCAAG-3´, LSU1200R: 5´-GCATAGTTCACCATCTTTCGG-3´). For a single species, *Echinorhynchus cinctulus*, the 1600-bp fragment could not be amplified in full, but a partial 750-bp fragment was obtained by amplification and sequencing with the LSU5 and ECD2 primers. Amplification was done in 50 µl PCR reactions containing 200 µM of each deoxynucleotide, 2 mM MgCl_2_, 1 × reaction buffer (Perkin-Elmer, UK), 1 unit of *Taq* DNA polymerase (Amplitaq, Perkin-Elmer, UK), 10 pM of each primer and c.200 ng template DNA. Thermal cycling involved an initial denaturation of 95 °C for 5 minutes followed by 30 cycles of 94 °C/1 minute, 50 °C/1 minute and 72 °C/1 minute, and a final incubation at 72 °C/5 minutes. A minimum of two successful reactions were performed for each template. Amplified products were run on a 1% TAE agarose gel, cut out, pooled and purified using a QIAquick PCR Purification Kit (QIAGEN). Sequencing was performed with standard procedures on a 373 ABI automated sequencer with the ABI PRISM TM dye terminator cycle sequencing ready reaction kit (Perkin-Elmer, UK).The sequences were aligned using ClustalW (Thompson et al. 1994) with default weighting and gap penalties.

For analysis of a part of the mitochondrial COI gene, the universal “barcoding” primers of Folmer et al. (1994) were used for amplification and sequencing, following the procedures in Väinölä et al. (2001). The final COI alignment used for analyses was 585 bp long.

### Phylogenetic analysis

The 28S rDNA and COI sequences were analyzed independently and also concatenated into a single dataset. Three methods of phylogenetic reconstruction were applied to each dataset: Bayesian inference (BI), maximum likelihood (ML) and maximum parsimony (MP). *Acanthocephalus lucii* was used as an outgroup in all analyses. For the phylogenetic reconstruction methods involving modelling of sequence evolution (BI and ML), the data-sets were partitioned to accommodate heterogeneity in patterns and rates of substitutions between genes and/or codon positions. The COI data-set was divided into three partitions, one for each codon position. The concatenated 28S rDNA and COI data-set was separated into four partitions, one for the 28S rDNA sequence and three for each of the codon positions in the COI sequence. The 28S rDNA data-set was not partitioned.

Mr Bayes version 3.2.2 (Huelsenbeck and Ronquist 2001, Ronquist and Huelsenbeck 2003) was used for BI, with the following settings: two simultaneous runs with four Markov chains (one cold and three heated) and one million MCMC generations, sampled every 500 generations and a temperature parameter of 0.1. To avoid the uncertainty of selecting the correct substitution model *a priori*, reversible jump MCMC was used to sample across all possible time-reversible rate matrices according to their posterior probability (Ronquist et al. 2012). For each run log likelihood was plotted against number of generations and burn-in was assumed to have occurred when the curve reached a plateau. The number of generations (samples) discarded as burn-in were 10,000 (20), 30,000 (60) and 70,000 (40), for the 28S rDNA, COI and concatenated data-sets, respectively.

ML analysis was carried out using the genetic algorithm implemented in MetaPIGA 3.1 (Helaers and Milinkovitch 2010). The nucleotide substition model for each data-set was selected using the Bayesian Information Criterion (BIC). For the 28S rDNA data-set, the generalized time reversible (GTR) model (Tavaré 1986) with gamma distributed rate heterogeneity (four categories) was chosen. TN93 (Tamura and Nei 1993) and a gamma distribution with four rate categories was selected as the best model for the COI and concatenated 28S rDNA + COI data-sets (further details of model parameters in Suppl. material 2). Each analysis was run with a minimum of 100 and a maximum of 10,000 replicates and was stopped once the mean relative error among 10 consecutive consensus trees was less than 5%. Starting trees were generated by loose neighbour joining and were selected using the tournament algorithm.

MP analysis was performed using PAUP version 4.0b10 (Swofford 2003). Gaps in the 28S rDNA sequence alignments were treated as missing data. An exhaustive search was performed on each data-set and the frequency distribution of tree scores was determined. Bootstrap resampling (n = 10,000) was used with the branch and bound algorithm to quantify clade support.

Phylograms and other graphics were created using R (R Core Team 2014) and the APE package (Paradis et al. 2004).

## Data resources

All sequence data have been submitted to GenBank; accession numbers are provided in Table 1. Additionally, the sequence alignment used in this study is provided in Suppl. material 1.

## Results

### Patterns of sequence divergence

The aligned partial 28S rDNA sequence data consisted of 1,607 nucleotide sites for all taxa except *Echinorhynchus cinctulus*, for which only the first 750 base pairs of the segment could be sequenced (Suppl. material 1). In comparisons among the *Echinorhynchus* sequences, 261 (16.2%) of the 1,607 sites were variable, and 133 of those (51%) were parsimony informative. Of the ingroup taxa, *Echinorhynchus salmonis* and *Echinorhynchus cinctulus* sequences were the most divergent, differing by 15% and 7%, respectively, from the remaining group of very closely related sequences, which only differed by less than 1% from each other. Five samples possessed identical 28S sequences: *Echinorhynchus gadi* sp. I (Baltic Sea), *Echinorhynchus gadi* sp. I (White Sea), *Echinorhynchus gadi* sp. III, *Echinorhynchus bothniensis* and *Echinorhynchus ‘bothniensis’* (Table 3).

**Table 3.** Observed sequence divergence (%) between pairs of echinorhynchid species for the 28S rDNA (below the diagonal) and COI sequence data (above the diagonal).

	1	2	3	4	5	6	7	8	9	10
**1. *Acanthocephalus lucii***	—	36.1	33.3	34.5	32.8	34.2	34.4	34.0	34.0	34.7
**2. *Echinorhynchus salmonis***	18.5	—	29.7	27.7	28.7	29.7	29.7	29.4	28.7	28.9
**3. *Echinorhynchus cinctulus***	31.1	23.1	—	21.7	22.2	21.5	21.7	22.9	22.9	23.1
**4. *Echinorhynchus brayi***	19.1	15.5	6.6	—	16.8	17.4	17.3	19.0	17.1	18.0
**5. *Echinorhynchus truttae***	19.3	15.3	7.5	0.8	—	8.2	8.4	9.1	8.9	8.9
**6. *Echinorhynchus gadi* sp. I (Baltic Sea)**	19.2	15.4	7.1	0.5	0.3	—	0.2	7.2	6.5	6.3
**7. *Echinorhynchus gadi* sp. I (White Sea)**	19.2	15.4	7.1	0.5	0.3	0.0*	—	7.4	6.5	6.3
**8. *Echinorhynchus gadi* sp. III**	19.2	15.4	7.1	0.5	0.3	0.0*	0.0*	—	3.3	3.1
**9. *Echinorhynchus bothniensis***	19.2	15.4	7.1	0.5	0.3	0.0*	0.0*	0.0*	—	1.5
**10. *Echinorhynchus 'bothniensis'***	19.2	15.4	7.1	0.5	0.3	0.0*	0.0*	0.0*	0.0*	—

* sequences are identical

In the 585 base-pair alignment of the COI sequences, 249 (42.6%) of the nucleotide sites were variable within *Echinorhynchus*, of which 62 (24.9%) were at a first codon position, 23 (9.2%) at a second codon position and 164 (65.9%) at a third codon position (Suppl. material 1). Of the variable sites, 148 (59.4%) were parsimony informative. Uncorrected sequence divergence between pairs of *Echinorhynchus* sequences ranged from 0.2% (Baltic vs. White Sea sequences of *Echinorhynchus gadi* sp. I) to 29.7% (*Echinorhynchus salmonis* vs. *Echinorhynchus cinctulus* and *Echinorhynchus salmonis* vs. *Echinorhynchus gadi* sp. I) (Table 3). In pairwise comparisons of samples with relatively similar COI sequences (uncorrected sequence divergence < 20%), most substitutions were transitions (Suppl. materials 3, 4). However, in comparisons involving the more divergent *Echinorhynchus cinctulus*, *Echinorhynchus salmonis* and *Acanthocephalus lucii*, transitions were generally outnumbered by transversions, suggesting that multiple substitutions at some variable nucleotide sites have erased the record of previous transitions. Saturation occurs primarily at the fast evolving third codon position (Suppl. materials 3, 4).

### Phylogenetic relationships

**Figure 3.**
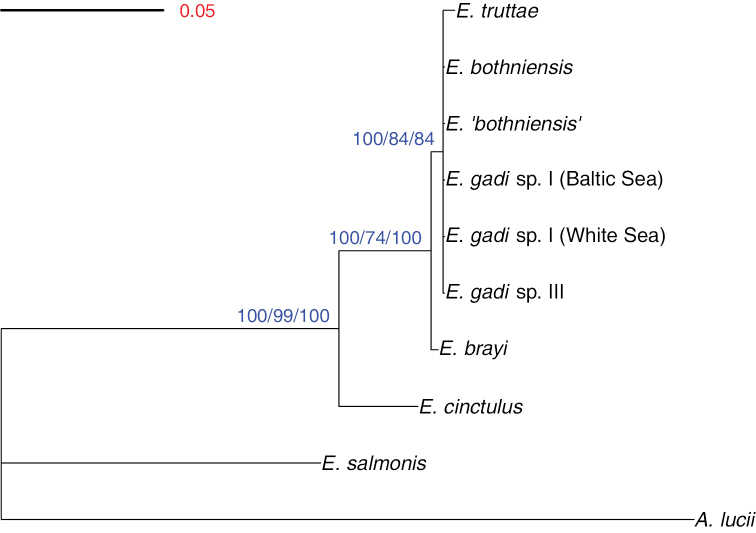
Phylogram estimated using Bayesian inference analysis of 28S rDNA sequence data. Numbers at nodes are clade support values (%) for each method of phylogeny reconstruction (BI/ML/MP). Tree is rooted on the outgroup *Acanthocephalus lucii*.

Since identical sequences were obtained from members of the *Echinorhynchus gadi* complex, *Echinorhynchus bothniensis* and *Echinorhynchus ‘bothniensis’*, the 28S rDNA data-set could only be used to resolve the deeper branches in the phylogeny. BI identified a hierarchy of three clades, each with a maximal posterior probability (Fig. 3): ((((*Echinorhynchus gadi* complex + *Echinorhynchus bothniensis* complex, *Echinorhynchus truttae*), *Echinorhynchus brayi*), *Echinorhynchus cinctulus*), *Echinorhynchus salmonis*). The 50% consensus tree derived from the ML analysis had an identical topology to the BI tree and moderate bootstrap support for each of the three clades (74–99%). MP analysis yielded two most parsimonious trees (length = 488, consistency index (CI) = 0.957, retention index (RI) = 0.859), the consensus cladogram for which also had an identical topology to the BI phylogram and provided strong bootstrap support (84–100%) for all three clades.

**Figure 4.**
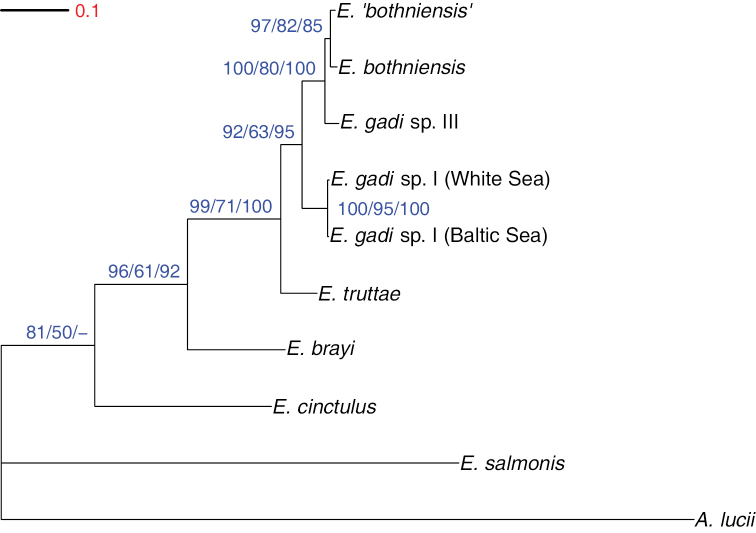
Phylogram estimated using Bayesian inference analysis of COI sequence data. Numbers at nodes are clade credibility values (%) for each method of phylogeny reconstruction (BI/ML/MP). Tree is rooted on the outgroup *Acanthocephalus lucii*.

A fully resolved tree was recovered from the mitochondrial COI data-set (Fig. 4). The topology for the basal parts was identical to that resolved by the 28S data above. Within the remaining terminal cluster of very closely related taxa, the *Echinorhynchus gadi* sp. I sequences from the two regions grouped together and so did *Echinorhynchus bothniensis* + *Echinorhynchus ‘bothniensis’*. *Echinorhynchus gadi* sp. III made a sister group to the *Echinorhynchus bothniensis* clade rather than to *Echinorhynchus gadi* sp. I. The BI analysis yielded high posterior probability values (92–100%) for all clades, except for the one comprising all *Echinorhynchus* spp. but *Echinorhynchus salmonis* (81%). The ML tree topology was identical to that from BI, but with a weaker clade support (50–95%).

**Figure 5.**
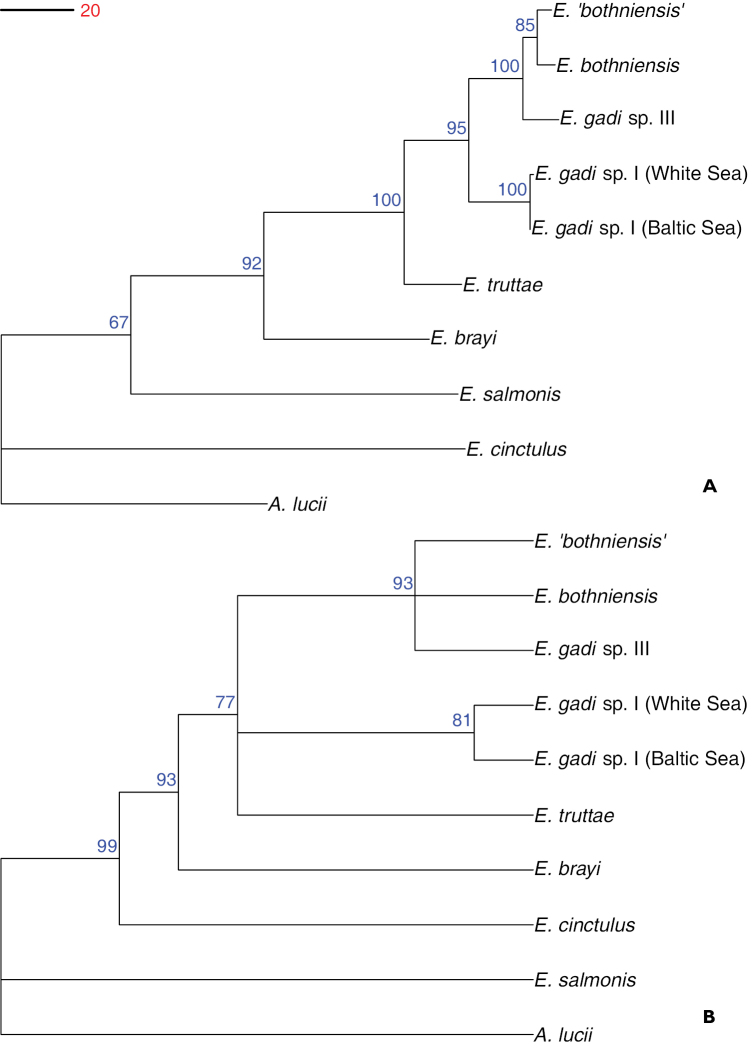
Phylogenetic relationships of *Echinorhynchus* spp. inferred from maximum parsimony analysis of COI data-set. Trees are rooted on the outgroup *Acanthocephalus lucii*. **A** Phylogram estimated using maximum parsimony analysis of COI sequence data. Numbers at nodes indicate bootstrap support (n = 10,000) **B** Consensus cladogram from maximum parsimony analysis of COI sequence data excluding third codon positions. Numbers at nodes indicate bootstrap support (n = 10,000).

MP analysis of the COI data-set produced a single most parsimonious tree, 542 steps long (CI = 0.795, RI = 0.615), which differed from the BI and ML phylograms at a single point, regarding the basal placement of *Echinorhynchus cinctulus* instead of *Echinorhynchus salmonis* (Fig. 5a). Strong bootstrap support (86–100%) was found for all clades, except for that defining the basal node and comprising all *Echinorhynchus* but *Echinorhynchus cinctulus*, which only had 66 % support. The conflict between the MP vs. the BI/ML trees appears to be the result of homoplasy at third codon positions. When MP analysis was repeated after eliminating the 3rd codon positions, a total of five most parsimonious trees (length = 177, CI = 0.932, RI = 0.786) were found. The consensus cladogram for these five trees (Fig. 5b) is concordant with the BI/ML tree for the full COI data-set. However, the relationships of the six most similar sequences were not fully resolved with the reduced 1st+2nd position data, which retained just 11 variable and only seven parsimony informative characters as regards information within the six-sequence cluster.

**Figure 6.**
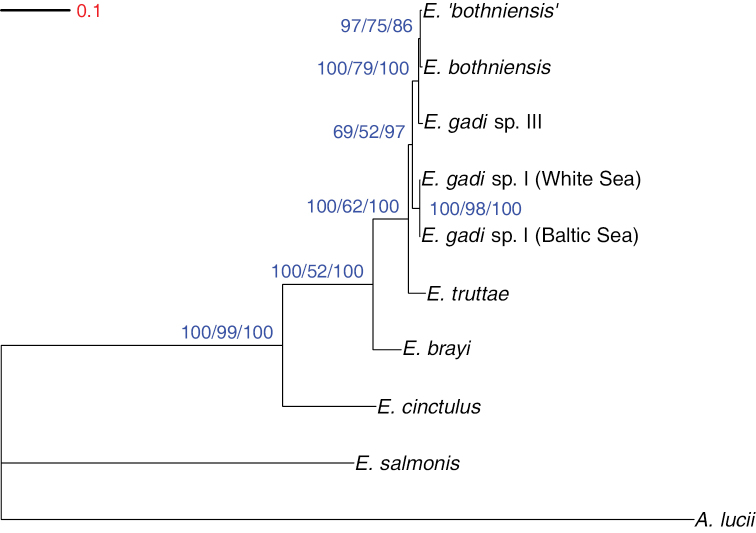
Phylogram estimated using Bayesian inference analysis of concatenated 28S rDNA and COI sequence data. Numbers at nodes are clade support values (%) for each method of phylogeny reconstruction (BI/ML/MP). Tree is rooted on the outgroup *Acanthocephalus lucii*.

BI, ML and MP analysis of the combined data-sets all yielded the same phylogram, which was topologically identical to the BI/ML tree for the COI data-set and displayed similar support for most clades (Fig. 6). The most parsimonious tree (CI = 0.869; RI = 0.691) had a length of 1,033 steps.

## Discussion

The following discussion is based on the fully resolved phylogeny recovered from the total molecular data. It is important to note that, whereas the deeper branches in the phylogeny are supported by sequence data from both genes, the interrelationships of the five most closely related species were resolved using the COI data-set alone.

### Systematics

**Figure 7.**
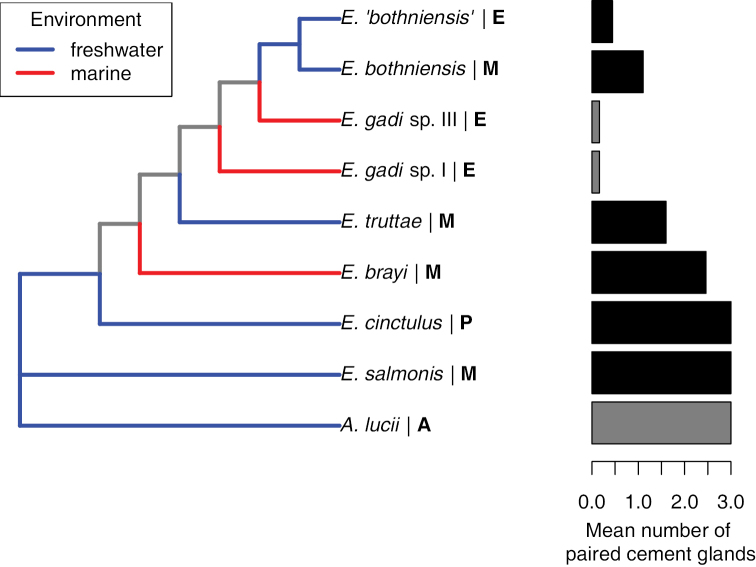
Aquatic environment (freshwater/marine) mapped on to the fully resolved phylogeny inferred from the concatenated 28S and COI sequences. Bold letter indicates genus according to Petrochenko’s (1956) scheme: E, *Echinorhynchus*; M, *Metechinorhynchus*; P, *Pseudoechinorhynchus*. The bar chart shows the mean number of paired cement glands in each taxon. Data for *Echinorhynchus* spp. are from Table 2. Since the particular cement gland pattern exhibited by each of the species of the *Echinorhynchus gadi* group is not known, data from a collection of worms determined as *Echinorhynchus gadi* have been used for *Echinorhynchus gadi* spp. I & III (the bars for these species are shaded grey rather than black, to indicate a lower level of confidence in the data). Since *Acanthocephalus lucii* typically displays paired cement glands (Petrochenko 1956), the mean number of paired cement glands in this taxon was assumed to be approximately three (bar shaded grey to indicate approximation).

No support for Petrochenko’s (1956) revision of *Echinorhynchus*, involving subdivision into three genera based on the cement gland pattern, is provided by the present study. The phylogeny derived from the total molecular data (Fig. 7) indicates that *Metechinorhynchus* (*sensu* Petrochenko 1956) may be a polyphyletic assemblage. Furthermore, *Echinorhynchus* (*sensu* Petrochenko 1956) would be paraphyletic, if evidence of cement gland differentiation in the *Echinorhynchus bothniensis* complex is deemed significant. Thus, this study supports the work of Väinölä et al. (1994), who rejected the hypothesis of monophyly of *Metechinorhynchus* on the basis of allozyme data from a more limited range of taxa. In view of the poor morphological definition of Petrochenko’s genera and their incongruity with phylogenetic hypotheses from independent data-sets, we concur with other authors (Yamaguti 1963, Huffman and Kliever 1977, Amin and Redlin 1980, Amin 2013), who have recommended that the names *Metechinorhynchus* and *Pseudoechinorhynchus* should be designated junior synonyms of *Echinorhynchus*. Golvan (1994) relegated *Echinorhynchus* (*sensu* Petrochenko 1956), *Metechinorhynchus* and *Pseudoechinorhynchus* to the status of subgenera of *Echinorhynchus* (*sensu lato*). However, this scheme is subject to the same criticisms as Petrochenko’s original classification and so should also be dismissed.

Cement gland arrangement displays continuous variation, from the pattern of three regular pairs through to the strictly moniliform pattern, with each *Echinorhynchus* species displaying a range of variation along this continuum (Table 2). The absence of discrete character states presents practical difficulties in using cement gland arrangement as a criterion of generic identity. To examine the presence of a phylogenetic signal in cement gland pattern, we used the average number of paired cement glands in each species as a summarizing variable, and plotted the variation of this character alongside the fully resolved tree (Fig. 7). Since cement gland patterns have not been determined for any of the electrophoretically identified species of the *Echinorhynchus gadi* complex, *Echinorhynchus gadi* spp. I and III were assumed to display the same cement gland pattern recorded from unidentified specimens of the *Echinorhynchus gadi* complex from gadid fishes (Wayland 2002). On the phylogeny comprising of six nested clades, an association between the clade identity and the average number of paired cement glands is evident, indicating that cement gland pattern conveys a phylogenetic signal, although the variability implies much homoplasy also. A more rigorous test of this morphological character will require accurate data for the species of the *Echinorhynchus gadi* complex. Notably, the species on the basal branches of the phylogeny (*Echinorhynchus salmonis* and *Echinorhynchus cinctulus*) displayed three pairs of cement glands, suggesting that this pattern is the plesiomorphic condition.

Further and more conclusive evidence that the ancestral cement gland arrangement is three regular pairs is available from both outgroup comparison and ontogeny. Firstly, outgroup comparison is based on the assumption that the character state found in related groups is the plesiomorphic condition (Watrous and Wheeler 1981). For the purposes of this comparison, genera in the same subfamily as *Echinorhynchus* have been chosen as outgroups. In the most recent classification of the Acanthocephala (Amin 2013), the Echinorhynchinae Cobbold, 1876 comprises six genera in addition to *Echinorhynchus*, namely *Acanthocephalus* Koelreuther, 1771, *Anuracanthorhynchus* Bursey, Vreibradic, Hatano & Rocha, 2006, *Brasacanthus* Thatcher, 2001, *Frilloechinorhynchus* Bhattacharya, 2007, *Pilum* Williams, 1976 and *Pseudoacanthocephalus* Petrochenko, 1956. *Acanthocephalus* and *Pseudoacanthocephalus* are diverse, containing 53 and 18 species respectively; the other four genera are monotypic. The majority of the species in these outgroup genera display regular pairs of cement glands, indicating that this is the plesiomorphic condition. Three regular pairs of cement glands are typical of the many species of *Acanthocephalus* and *Pseudoacanthocephalus*, whereas the monotypic *Pilum* is characterized by four regular pairs (Petrochenko 1956, Williams 1976). *Anuracanthorhynchus tritaxisentis* Bursey, Vreibradic, Hatano & Rocha, 2006 and *Brasacanthus sphoeroides* Thatcher, 2001, the type-species and sole representatives of their respective genera, have their cement glands arranged in parallel, a pattern not found in *Echinorhynchus* (see Thatcher 2001, Bursey et al. 2006). The only species in the outgroup to display its six cement glands in the moniliform pattern is *Frilloechinorhynchus meyeri* (Gupta & Naqvi, 1986) (see Bhattacharya 2007). Ontogenic evidence comes from a study of the embryology of *Echinorhynchus truttae*, in which the developing cement gland primordia were illustrated as three, approximately regular, pairs (see figure 7 and 8 of Awachie 1966); as an adult *Echinorhynchus truttae* never displays three regular pairs of cement glands (Table 2). Thus, the moniliform pattern represents a derived or apomorphic condition.

**Figure 8.**
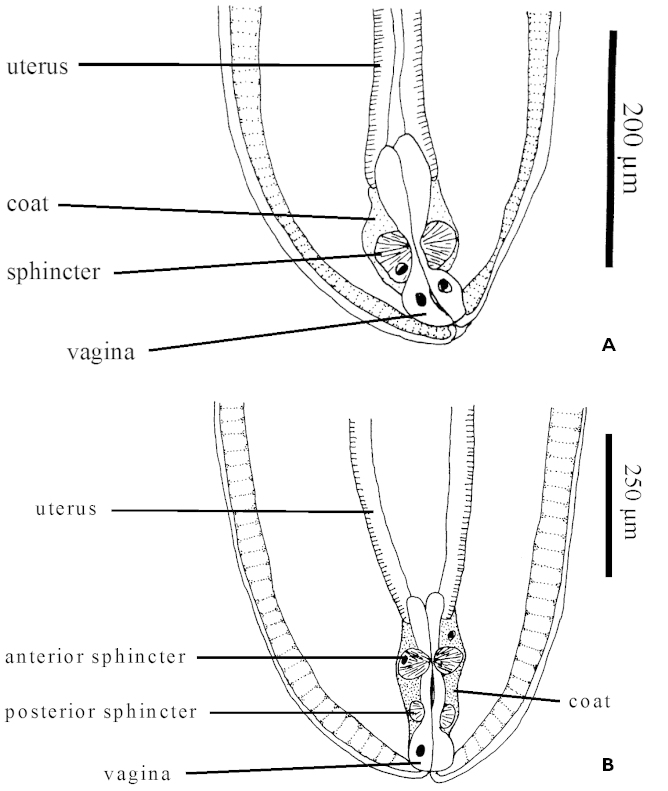
Structure of the vagina in *Echinorhynchus* spp. **A** *Echinorhynchus brayi*, a species with a single vaginal sphincter **B** *Echinorhynchus salmonis*, a species with two vaginal sphincters.

*Echinorhynchus cinctulus* and *Echinorhynchus salmonis* exhibit a relatively strong genetic divergence from each other and from the other taxa of the ingroup (Table 3). Each of these taxa also displays physical peculiarities not observed in other members of the ingroup. A study of the morphology of the reproductive system of *Echinorhynchus* spp. (Wayland 2002) revealed that female *Echinorhynchus salmonis* possess two vaginal sphincters, whereas all of the other taxa in the ingroup have a single vaginal sphincter (Fig. 8). Since the outgroup used in the current analysis, *Acanthocephalus lucii*, also has only a single vaginal sphincter, the double vaginal spincter may represent an apomorphy.

The acanthors of *Echinorhynchus cinctulus* display a unique pattern of hooks and spines which has not been observed in other species of *Echinorhynchus*, although relatively few taxa have been studied (Grabda-Kazubska 1964). The acanthors of *Echinorhynchus gadi* and *Echinorhynchus truttae* exhibit a well differentiated armature consisting of two large spade-like hooks and other smaller hooks on the rostellum plus small spines covering the rest of the body (Grabda-Kazubska 1964). Acanthors of *Echinorhynchus bothniensis*, *Echinorhynchus ‘bothniensis’*, *Echinorhynchus brayi* and *Echinorhynchus salmonis* display a similar armature (Wayland 2002). In contrast, the relatively undifferentiated armature of the acanthors of *Echinorhynchus cinctulus* comprises small hooks on the rostellum and small spines covering the rest of the body (Grabda-Kazubska 1964, Grabda-Kazubska and Ejsymont 1969). The acanthors of the outgroup taxon, *Acanthocephalus lucii*, display a well differentiated, but asymmetrical, armature (Grabda-Kazubska 1964). While neither the type of acanthor armature nor the number of vaginal sphincters provide synapomorphies for clades identified in this study, these characters may yet prove to be useful in a revision of the genus.

Another taxonomic finding of the current study is paraphyly of the *Echinorhynchus gadi* group with respect to the monophyletic *Echinorhynchus bothniensis* group (Fig. 7). Thus, the current terminology is misleading, as it seems to imply that *Echinorhynchus gadi* and *Echinorhynchus bothniensis* are distinct groups (clades), when in fact *Echinorhynchus bothniensis* is a subgroup nested within the *Echinorhynchus gadi* species group. At this point, these informal taxonomic labels may however be maintained, as they convey biological information related to the habitat and host spectrum of the taxa. The *Echinorhynchus gadi* group parasitize fish and amphipods in the sea, whereas the *Echinorhynchus bothniensis* group infect fish and *Mysis* spp. in fresh and brackish waters.

One significant problem in the systematics of *Echinorhynchus*, which could not be addressed with the current data, is the monophyly of the genus. Further phylogenetic analyses incorporating a range of echinorhynchid acanthocephalans will be needed to resolve this issue. The relatively slowly evolving 28S rRNA gene, along with nuclear protein coding genes, should prove to be particularly useful in this respect.

### Zoogeography

Since our phylogeny represents only a small proportion of the species in the genus, it is impossible to make any definitive claims about the zoogeography of this group of worms. However, the limited observations do suggest hypotheses that could be tested with additional data.

*Echinorhynchus* spp. are distributed from the Arctic (e.g. Shostak et al. 1986) to the Antarctic (e.g. Zdzitowiecki 1986), occurring in most aquatic environments, including mountain streams, rivers, lakes, estuaries, coastal marine waters and the deep-sea. They are found in both temperate and tropical regions (e.g. Machado Filho 1948). No other genus of acanthocephalans is known to display such an extensive geographical range. The genus may have had its origins in freshwater, because taxa displaying what is postulated to be the plesiomorphic cement gland arrangement (three regular pairs) occur almost exclusively in freshwater fishes, whereas the apomorphic condition (moniliform pattern) is generally only found in marine species. Transitional forms in the assumed transformation from regular pairing of cement glands to the moniliform pattern can be found in freshwater and the sea. Furthermore, of the six other genera of the subfamily Echinorhynchinae, four (including the species-rich *Acanthocephalus* and *Pseudoacanthocephalus*) are composed entirely of parasites of freshwater fish or amphibians (Petrochenko 1956, Yamaguti 1963, Williams 1976, Bursey et al. 2006). Basal positions in the molecular phylogeny for two of the freshwater species (*Echinorhynchus salmonis* and *Echinorhynchus cinctulus*) lend additional support to this hypothesis. However, implicit in this supposition is the unverified assumption of a monophyletic *Echinorhynchus*.

From this suggested freshwater origin and radiation, *Echinorhynchus* spp. have invaded the sea, most likely several times (Fig. 7). Various scenarios may have facilitated the colonisation of marine hosts. Of particular relevance in this respect is the association of *Echinorhynchus* spp. with diadromous definitive hosts. Fish hosts of *Echinorhynchus* spp. which migrate between freshwaters and the sea include *Coregonus lavaretus* (L.), *Osmerus eperlanus* (L.), *Salmo salar* L. and *Salmo trutta* L. (see Kottelat 1997). Estuaries and other brackish environments, such as the Bothnian Bay, Baltic Sea, may provide further opportunities for parasite exchange between freshwater and marine fish. The Bothnian Bay has a very low salinity (less than 0.3%) and so its fish fauna is dominated by species of freshwater origin. Nevertheless, marine fishes, such as *Gadus morhua* L., occasionally enter this region, presumably following more saline currents from the main region of the Baltic Sea (Valtonen and Crompton 1990). Acanthocephalans display a relatively weak specificity towards their definitive hosts (Golvan 1957), a phenomenon favouring host-switching (García-Varela et al. 2013). The adoption of new definitive hosts would potentially allow *Echinorhynchus* spp. to invade new aquatic habitats and so be an important factor in geographical range extension. Moreover, gradual adaptation of species of freshwater origin to marine conditions (and *vice versa*) might take place in brackish environments, such as estuaries.

Evidence of a re-invasion of freshwater by marine stock can also be found in the fully resolved phylogeny (Fig. 7). The clade comprising the freshwater taxa *Echinorhynchus bothniensis* and *Echinorhynchus ‘bothniensis’* is nested within the clade for the species of the closely related, but marine, *Echinorhynchus gadi* group. Thus *Echinorhynchus bothniensis* and *Echinorhynchus ‘bothniensis’* represent either: (1) the result of two independent invasions of freshwater from marine stock; or (2) the outcome of invasion of freshwater by a single lineage of marine origin, followed by divergence within fresh or brackish waters. The latter hypothesis seems more likely since the *Echinorhynchus bothniensis* group taxa are thought to have co-speciated with their intermediate hosts, i.e. freshwater/brackish species of the *Mysis relicta* species group (Väinölä et al. 1994). The definitive hosts of the *Echinorhynchus bothniensis* group include several diadromous species, such as *Salmo trutta*, *Osmerus eperlanus* and *Platichthys flesus* (see Valtonen and Crompton 1990). Such euryhaline species were probably instrumental in carrying the common ancestor from the sea into inland waters.

### Final comments

This preliminary investigation of the phylogenetic relationships within *Echinorhynchus* (*sensu lato*) underscores the argument for rejecting Petrochenko’s (1956) revision of the genus, by demonstrating that neither *Echinorhynchus* (*sensu* Petrochenko 1956) nor *Metechinorhynchus* represent natural monophyletic groups. Nevertheless, *Echinorhynchus* is a large and growing genus, and consequently its division into smaller units is desirable. A revision of this genus is beyond the scope of the current study and will require tandem molecular phylogenetic and morphological analyses of a much larger sample of taxa attributed to *Echinorhynchus* and to related genera. Such analyses would also provide additional insights into the factors determining the geographical distribution and host relationships of echinorhynchid acanthocephalans in general.

## Supplementary materials

Supplementary material 1 Aligned and concatenated partial sequences of COI and 28S rDNA Data type: Nexus file Explanation note: Aligned and concatenated partial sequences of COI and 28S rDNA in nexus format. Aligned partial sequences of COI and 28S rDNA from each acanthocephalan population have been concatenated. Gaps are indicated by ‘-’. The first 585 characters in each block correspond to COI and the remainder to 28S rDNA. The file contains data for all nine Echinorhynchus samples and the outgroup taxon, Acanthocephalus lucii. This nexus file was used in all phylogenetic analyses. This dataset is made available under the Open Database License (http://opendatacommons.org/licenses/odbl/1.0/). The Open Database License (ODbL) is a license agreement intended to allow users to freely share, modify, and use this Dataset while maintaining this same freedom for others, provided that the original source and author(s) are credited.Matthew T. Wayland, Jouni K. Vainio, David I. Gibson, Elisabeth A. Herniou, D. Timothy J. Littlewood, Risto Väinölä

Supplementary material 2 Maximum likelihood model parameters Data type: Adobe PDF file Explanation note: Model parameters used in the maximum likelihood approach to phylogenetic reconstruction. This dataset is made available under the Open Database License (http://opendatacommons.org/licenses/odbl/1.0/). The Open Database License (ODbL) is a license agreement intended to allow users to freely share, modify, and use this Dataset while maintaining this same freedom for others, provided that the original source and author(s) are credited.Matthew T. Wayland, Jouni K. Vainio, David I. Gibson, Elisabeth A. Herniou, D. Timothy J. Littlewood, Risto Väinölä

Supplementary material 3 Nucleotide substitutions Data type: Comma-separated-value file of measurements Explanation note: Substitutions of nucleotides (transitions/transversions) for 28S rDNA (below the diagonal) and COI sequence data (above the diagonal). This dataset is made available under the Open Database License (http://opendatacommons.org/licenses/odbl/1.0/). The Open Database License (ODbL) is a license agreement intended to allow users to freely share, modify, and use this Dataset while maintaining this same freedom for others, provided that the original source and author(s) are credited.Matthew T. Wayland, Jouni K. Vainio, David I. Gibson, Elisabeth A. Herniou, D. Timothy J. Littlewood, Risto Väinölä

Supplementary material 4 Patterns of COI sequence variation Data type: Adobe PDF file Explanation note: Patterns of COI sequence variation. Graphs and discussion of patterns of nucleotide substitions in the COI data-set. This dataset is made available under the Open Database License (http://opendatacommons.org/licenses/odbl/1.0/). The Open Database License (ODbL) is a license agreement intended to allow users to freely share, modify, and use this Dataset while maintaining this same freedom for others, provided that the original source and author(s) are credited.Matthew T. Wayland, Jouni K. Vainio, David I. Gibson, Elisabeth A. Herniou, D. Timothy J. Littlewood, Risto Väinölä
