# Supplementary material for: The systematics of Echinorhynchus Zoega in Müller, 1776 (Acanthocephala, Echinorhynchidae) elucidated by nuclear and mitochondrial sequence data from eight European taxa
Source: Zookeys. 2015 Feb 26;(484):25–52. doi: 10.3897/zookeys.484.9132 (PMC4361782; doi:10.3897/zookeys.484.9132)
Supplement: Supplementary material 2 — Maximum likelihood model parameters [file zookeys-484-025-s002.pdf]

# Maximum Likelihood Model Parameters

The nucleotide substitution model for each data-set was selected using the Bayesian Information Criterion (BIC).

## 28S rRNA data-set

GTR model with gamma distributed rate heterogeneity (four categories). Rate matrix parameters were Alpha (0.488), Beta (3.648), Gamma (1.951), Delta (0.228) and Epsilon (5.938). Shape of the gamma distribution was 0.339 and there were zero invariant sites.

## CO1 data-set

TN93 model with gamma distributed rate heterogeneity (four categories).

Model parameters specific to each partition (codon position):

| Parameter | 1st | 2nd | 3rd |
|---|---|---|---|
| Transition between purines | 1.718 | 7.761 | 3.142 |
| Transition between pyrimidines | 6.733 | 0.613 | 4.359 |
| Shape of gamma distribution | 0.763 | 0.192 | 0.871 |
| Proportion of invariant sites (%) | 0 | 0 | 0 |

## Concatenated 28S rRNA + CO1 data-sets

TN93 model with gamma distributed rate heterogeneity (four categories).

Model parameters specific to each partition:

| Parameter | 28S rRNA | CO1: 1st | CO1: 2nd | CO1: 3rd |
|---|---|---|---|---|
| Transition between purines | 2.942 | 2.071 | 5.678 | 3.439 |
| Transition between pyrimidines | 5.763 | 8.216 | 0.706 | 4.850 |
| Shape of gamma distribution | 0.392 | 0.471 | 0.177 | 0.333 |
| Proportion of invariant sites (%) | 0 | 0 | 0 | 0 |
