# Supplementary material for: The systematics of Echinorhynchus Zoega in Müller, 1776 (Acanthocephala, Echinorhynchidae) elucidated by nuclear and mitochondrial sequence data from eight European taxa
Source: Zookeys. 2015 Feb 26;(484):25–52. doi: 10.3897/zookeys.484.9132 (PMC4361782; doi:10.3897/zookeys.484.9132)
Supplement: Supplementary material 4 — Patterns of COI sequence variation [file zookeys-484-025-s004.pdf]

Patterns of CO1 sequence variation

In comparisons between the 585 base-pair partial sequences for each *Echinorhynchus* sample, 249 (42.6%) nucleotide sites were variable, of which 62 (24.9%) were at a first codon position, 23 (9.2%) at a second codon position and 164 (65.9%) at a third codon position (Suppl. material 1). Of the 249 variable sites, 148 (59.4%) were parsimony informative, with 28 (18.9%) at a first codon position, 4 (2.7%) at a second codon position and 116 (78.4%) at a third codon position. Uncorrected sequence divergence between pairs of *Echinorhynchus* samples ranged from 0.2% (Baltic Sea population of *E. gadi* sp. I vs White Sea population) to 29.7% (*E. salmonis vs E. cinctulus* and *E. salmonis vs E. gadi* sp. I) .

In pairwise comparisons of samples with similar CO1 sequences (uncorrected sequence divergence <20%; i.e. pairs including any samples except *E. cinctulus, E. salmonis* and the outgroup, *A. lucii*), a strong bias of transitions to transversions was observed (Suppl. Material 2). However, in pairwise comparisons involving the more divergent taxa (*i.e. E. cinctulus, E. salmonis* and *A. lucii*), transitions were generally outnumbered by transversions. This observation suggested that multiple substitutions might have occurred at some variable nucleotide positions, erasing the record of previous transitions. The problem of saturation effects was investigated graphically (Figs 1,2,3). An approximately linear relationship between (i) transitions and transversions (Fig. 1a, b), (ii) transitions and sequence divergence (Fig. 2a, b), and (iii) transversions and sequence divergence (Fig. 3a, b), for all pairwise comparisons, demonstrates a relative lack of multiple substitutions (saturation effects) at the first and second codon positions.

At the third codon positions, a linear relationship between transitions and transversions could be demonstrated for pairwise comparisons excluding *E. cinctulus, E. salmonis* and *A. lucii* (Fig. 1c), with an observed transition to transversion ratio of approximately 1.75:1. However, pairwise comparisons of third codon positions involving the three most divergent taxa deviated markedly from this linear relationship and generally had a transition to transversion ratio which was close to unity. For this subset of taxa, the number of transitions and transversions at codon position three was uncorrelated (R^2 = 0.00008). The absence of bias in the transition to transversion ratio is interpreted as a saturation effect. Evidence of saturation at the third codon positions can also be seen when transitions or transversions are plotted against uncorrected sequence divergence; pairwise comparisons involving *E. cinctulus, E. salmonis* or *A. lucii* show fewer transitions (Fig. 2c), but more transversions than expected (Fig. 3c). This apparent homoplasy at the third codon positions means that caution is required when using the CO1 data-set to resolve the deeper branches of the *Echinorhynchus* phylogeny.

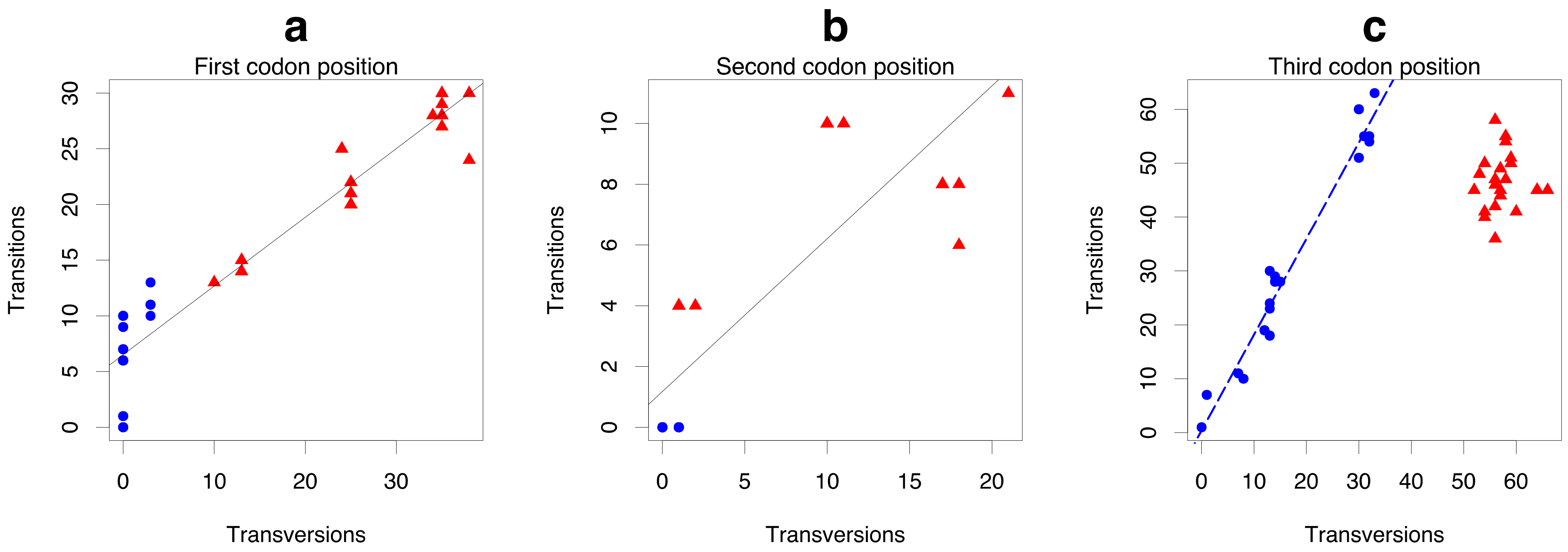

Figure 1.
Patterns of nucleotide substitutions at each codon position in the CO1 sequences. Graphs show the ratio of transitions (Ti) to transversions (Tv) in pairwise sequence comparisons. Comparisons involving *Acanthocephalus lucii, Echinorhynchus salmonis* or *E. cinctulus* are represented by triangles; comparisons not involving these taxa are represented by circles.
a: Ratio of Ti to Tv in pairwise sequence comparisons at codon position one. Equation of regression line: Ti = 0.617 x Tv + 6.514. Coefficient of determination (R^2) = 0.916.
b: Ratio of Ti to Tv in pairwise sequence comparisons at codon position two. Equation of regression line: Ti = 0.503 x Tv + 1.17. R^2 = 0.718
c: Ratio of Ti to Tv in pairwise sequence comparisons at codon position three. Pairwise comparisons not involving *A. lucii, E. cinctulus* or *E. salmonis* (blue circles) show a strong linear relationship (R^2 = 0.961) which can be described by the regression line (blue dashed line): Ti = 1.781 x Tv + 0.362. Pairwise comparisons involving *A. lucii, E. cinctulus* or *E. salmonis* (red triangles) do not show a linear relationship between Ti and Tv (R^2 = 0.0000814).

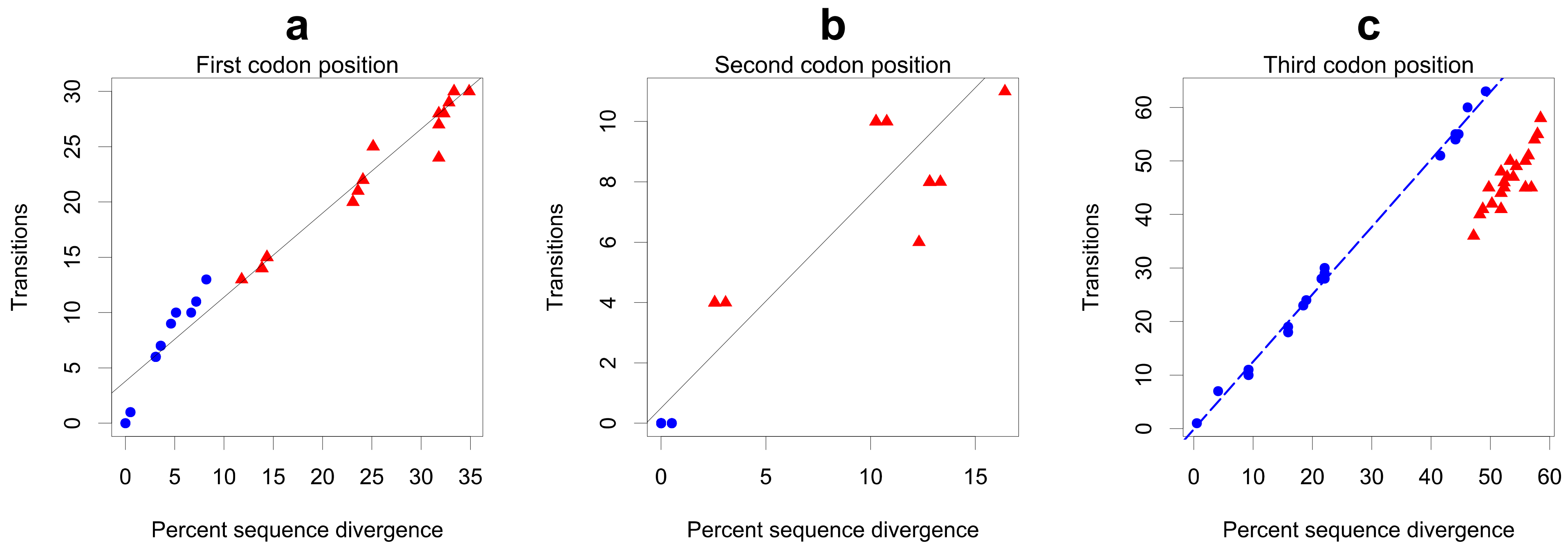

Figure 2.
Transitions and sequence divergence in CO1. Graphs show ratio of transitions (Ti) to uncorrected sequence divergence (D) in pairwise sequence comparisons. Comparisons involving *Acanthocephalus lucii, Echinorhynchus salmonis* or *E.cinctulus* are represented by triangles; comparisons not involving these taxa are represented by circles.
a: Ratio of Ti to D in pairwise sequence comparisons at codon position one. Equation of regression line: Ti = 0.760 x D + 3.788. Coefficient of determination (R^2) = 0.968.
b: Ratio of Ti to D in pairwise sequence comparisons at codon position two. Equation of regression line: Ti = 0.708 x D + 0.507. R^2 = 0.880.
c: Ratio of Ti to D in pairwise sequence comparisons at codon position three. Pairwise comparisons not involving *A. lucii, E. cinctulus* or *E. salmonis* (blue circles) show a strong linear relationship (R^2 = 0.995) which can be described by the regression line: Ti = 1.260 x D – 0.145. Pairwise comparisons involving *A. lucii, E. cinctulus* or *E. salmonis* (red triangles) do not show such a strong relationship (R^2 = 0.676); Ti = 1.311 x D – 23.475.

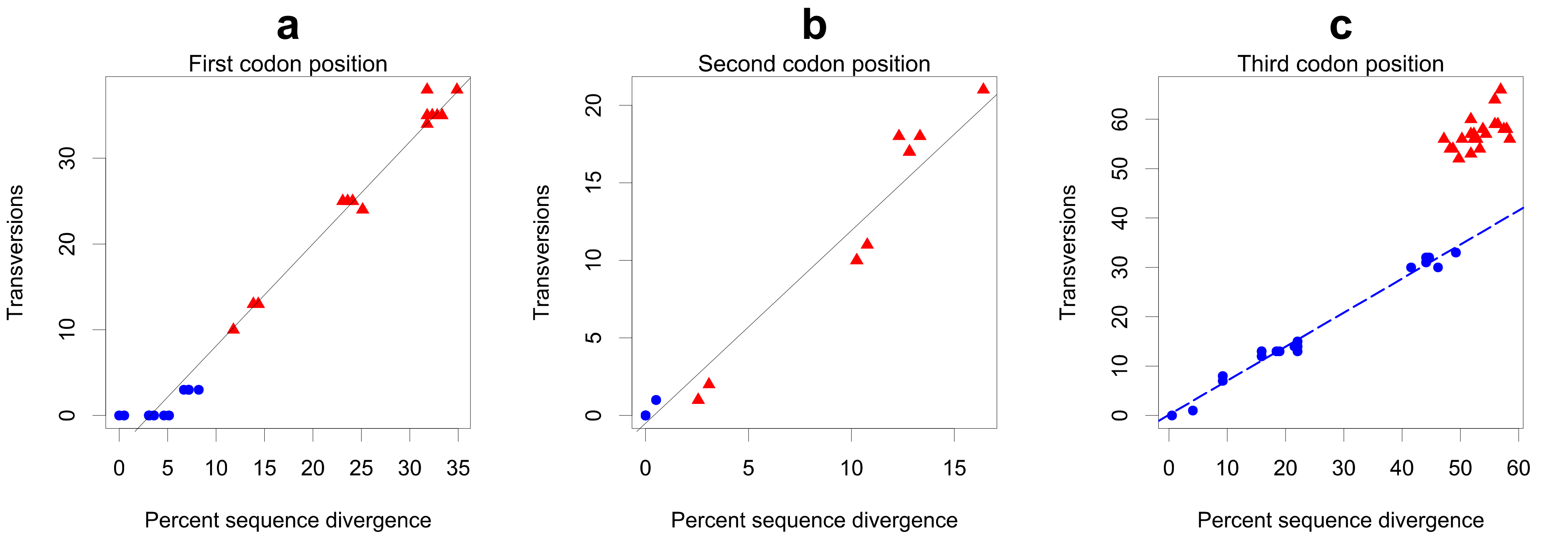

Figure 3.
Transversions and sequence divergence in CO1. Graphs show ratio of transversions (Tv) to uncorrected sequence divergence (D) in pairwise sequence comparisons. Comparisons involving *Acanthocephalus lucii, Echinorhynchus salmonis* or *E.cinctulus* are represented by triangles; comparisons not involving these taxa are represented by circles.
a: Ratio of Tv to D in pairwise sequence comparisons at codon position one. Equation of regression line: Tv = 1.190 x D – 3.791. R^2 = 0.987
b: Ratio of Tv to D in pairwise sequence comparisons at codon position two. Equation of regression line: Tv = 1.242 x D – 0.505. R^2 = 0.958.
c: Ratio of Tv to D in pairwise sequence comparisons at codon position three. Pairwise comparisons not involving *A. lucii, E. cinctulus* or *E. salmonis* (blue circles) show a strong linear relationship (R^2 = 0.984) which can be described by the regression line: Tv = 0.690 x D + 0.146. Pairwise comparisons involving *A. lucii, E. cinctulus* or *E. salmonis* (red triangles) do not show such a strong relationship (R^2 = 0.332); Tv = 0.639 x D + 23.482.
